# Prenatal Diagnosis of Williams-Beuren Syndrome Based on Suspected Fetal Hypotonia in Early Pregnancy

**DOI:** 10.7759/cureus.34841

**Published:** 2023-02-10

**Authors:** Nikolaos Tsagkas, Emmanouil Katsanevakis, Natalia Karagioti, Panagiotis Perdikaris, Michail Billis

**Affiliations:** 1 Obstetrics and Gynaecology, General Hospital of Agrinion, Agrinion, GRC; 2 Obstetrics and Gynaecology, United Lincolnshire Hospitals NHS Trust, Nottingham, GBR; 3 Fetal Medicine, Private Practice, Ioannina, GRC; 4 Obstetrics and Gynaecology, General University Hospital of Patras, Patras, GRC; 5 General Surgery, General Hospital of Lefkas, Lefkas, GRC

**Keywords:** prenatal genetic testing, fetal medicine specialist, first trimester scan, fetal hypotonia, williams-beuren syndrome

## Abstract

In this report, we describe a rare case of prenatal diagnosis of Williams-Beuren syndrome (WBS). While the prenatal diagnosis of WBS is very rare, in the current case, WBS was diagnosed in early pregnancy. The key element was the detection of fetal hands hypotonia and generalized fetal hypotonia at 17 weeks of gestation. This led to the diagnosis of WBS by molecular karyotyping, specifically array comparative genomic hybridization (arrayCGH) of the fetal DNA. The genetic material was acquired by extraction from the fetal cells which are abundant in the amniotic fluid drawn by amniocentesis. Clinical hypotonia of the affected individuals is a clinical characteristic that is widely associated with WBS; however, fetal hypotonia has not been described as a diagnostic criterion for the prenatal diagnosis of WBS.

## Introduction

The examination of the fetal anatomy by ultrasound in early pregnancy is invaluable because it can help with the early detection of fetal abnormalities. This could serve as the basis for informed choices and possibly early termination of pregnancy, which in many cases is the optimal choice. Early rather than late termination can be the optimal management in terms of women’s health, both physically and from a psychological perspective [1,2,3].

Williams-Beuren syndrome (WBS) is a chromosome microdeletion syndrome of a specific locus at chromosome 7 that includes the elastin (ELN) gene [4]. Its diagnosis is clinical and the first cases were described in the years 1961-1962 [5,6]. Individuals with WBS are diagnosed postnatally and a prenatal diagnosis is considered to be highly unlikely, albeit not impossible [7,8]. In this report, we present a rare case of prenatal diagnosis of WBS and we highlight the idea of prenatally diagnosing WBS based on suspected fetal hypotonia during a prenatal scan.

In the literature, studies on “normal fetal motility patterns” exist and the authors consider fetal motility as abnormal when immobility exceeds 13 minutes before 20 weeks of gestation [9].

## Case presentation

A primigravida aged 27 years with type 1 diabetes underwent a routine first-trimester scan by a fetal medicine specialist. The results of the first trimester tests are presented in Table 1.

**Table 1:** Summary of blood test results and ultrasound and karyotype findings WBS: Williams-Beuren syndrome; HCG: human chorionic gonadotropin: PAPPA: pregnancy-associated plasma protein A; NT: nuchal translucency

First-trimester biochemical test	Case WBS
Beta HCG	45.71IU/l, 1.262 MoM
PAPPA	7.710IU/l, 2.441 MoM
NT	1.5 mm
Gestational age at scan and findings	17 weeks of gestation, fetal hypotonia
Karyotype findings	Deletion of ~3,73 MB of the chromosomal region 7q11.22q11.23 (chr7:70,400,155-74,133,332)
Significance of findings	WBS (associated with cardiac anomalies, dysmorphic features, hypotonia, and neurodevelopmental delay)

Due to the history of diabetes, a follow-up scan was booked at 17 weeks of gestation for the optimal assessment of the fetal heart and the spinal cord. Anatomically, the fetus appeared normal but there was a strong impression of fetal hypotonia, more profound in the upper extremities. The movements of the fetal core, extremities, and fingers appeared to be fewer than expected for the specific week of gestation. Based on extensive scanning (duration: >4 hours, over two consecutive appointments) and after a detailed discussion with the parents, it was decided to perform an amniocentesis.

Fetal hypotonia is a nonspecific finding and a multidisciplinary team (MDT) approach was decided. The clinical geneticist in the MDT advised that the array comparative genomic hybridization (arrayCGH) analysis should be the method of choice in the presented case. Indeed, the arrayCGH analysis revealed a deletion of ~3,73 MB of the chromosomal region 7q11.22q11.23, where 30 genes are registered on the gene database OMIM (Online Mendelian Inheritance in Man), with the gene ELN being among them (*130160 in OMIM).

This specific finding is linked to WBS (#194050 in OMIM), which is characterized by cardiac anomalies, dysmorphic features, hypotonia, and neurodevelopmental impairment. A summary of the blood test results, ultrasound, and arrayCGH findings can be found in Table 1.

Following consultation, the couple opted for the termination of pregnancy. Termination was successfully performed by the “medical management pregnancy termination protocol”, which is based on the use of misoprostol tablets. The procedure was successful and uneventful; there was no need for admission to the surgical theater and no need for a blood transfusion. An en bloc fetoplacental specimen was produced and the woman was discharged home after spending 48 hours in the hospital.

## Discussion

In this report, we described a case of fetal hypotonia that led to the diagnosis of WBS early in pregnancy. The absence of fetal movements is considered abnormal when it exceeds 13 minutes before 20 weeks of pregnancy [9,10] and the nonspecific finding of reduced fetal motility/hands hypotonia, led to a rare identification and diagnosis of WBS prenatally. The incidence of the typical presentation of the syndrome is approximately 1/10,000 live births but atypical forms, of unknown incidence, have also been described [11].

In our literature search, we identified only nine cases of WBS that have been diagnosed prenatally (eight associated with deletion and one with duplication of genetic material). A summary of these studies and the indication for referral for invasive testing and molecular karyotyping are presented in Table 2.

**Table 2:** Literature search: cases of prenatal diagnosis of WBS WBS: Williams-Beuren syndrome; IUGR: intrauterine growth restriction; NT: nuchal translucency

Cases of prenatal diagnosis of WBS	Referral reason – indication for prenatal invasive testing	References
WBS deletion	IUGR 20 weeks of gestation	Marcato et al., 2013 [12]
WBS deletion	IUGR 32 weeks of gestation	Marcato et al., 2013 [12]
WBS deletion	Omphalocele 13 weeks of gestation → IUGR 18 weeks of gestation	Marcato et al., 2013 [12]
WBS deletion	Hydrops, polyhydramnios, absence of umbilical diastolic blood flow 30 weeks of gestation	von Dadelszen et al., 2000 [13]
WBS deletion	Ventricular septal defect 23 weeks of gestation	Kontos et al., 2008 [14]
WBS deletion	Symmetrical IUGR 20 weeks of gestation	Krzeminska et al., 2009 [7]
WBS deletion	IUGR 25 weeks of gestation	Popowski et al., 2011 [8]
WBS deletion	IUGR 33 weeks of gestation	Maeda et al., 2018 [15]
WBS duplication	Increased NT 13^+4 ^weeks of gestation, absence of nasal bone, inversion of a wave of ductus venosus	Marcato et al., 2013 [12]

WBS is usually diagnosed during childhood (median age: four years) and the diagnosis is clinical; the criteria include characteristic facial features that vary as per severity. Children often present with a flat nasal bridge, short upturned nose, periorbital puffiness, long philtrum, everted lower lip (dropping lower lip), widely spaced teeth, and delicate chin, whereas older patients have slightly coarse features, with full lips, wide mouth, and a full nasal tip [11].

**Figure 1:**
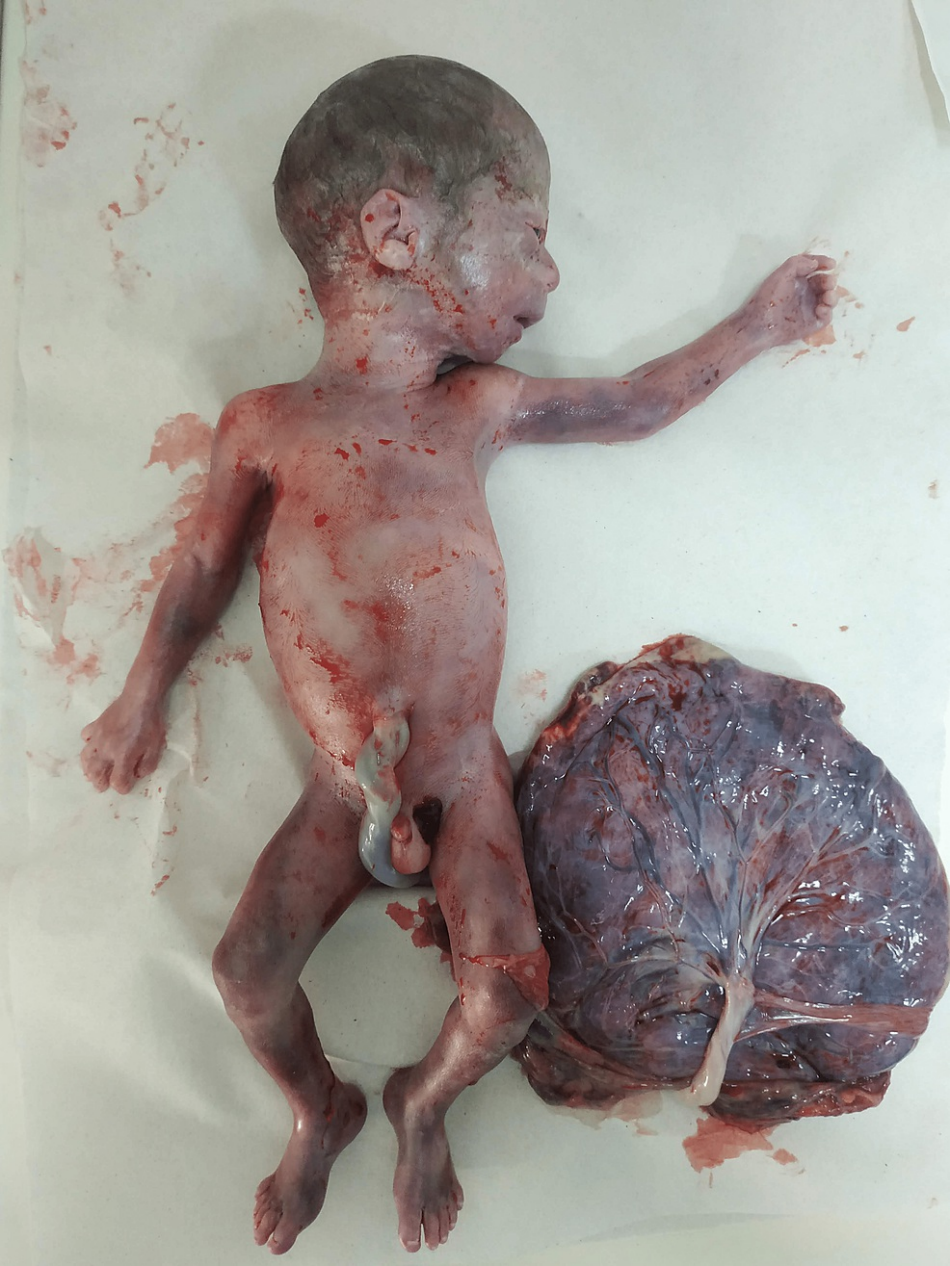
En bloc specimen: fetoplacental unit of the affected WBS fetus Personal archive of current case: Nikolaos Tsagkas MD, MSc; Consultant OB/GYN WBS: Williams-Beuren syndrome

**Figure 2:**
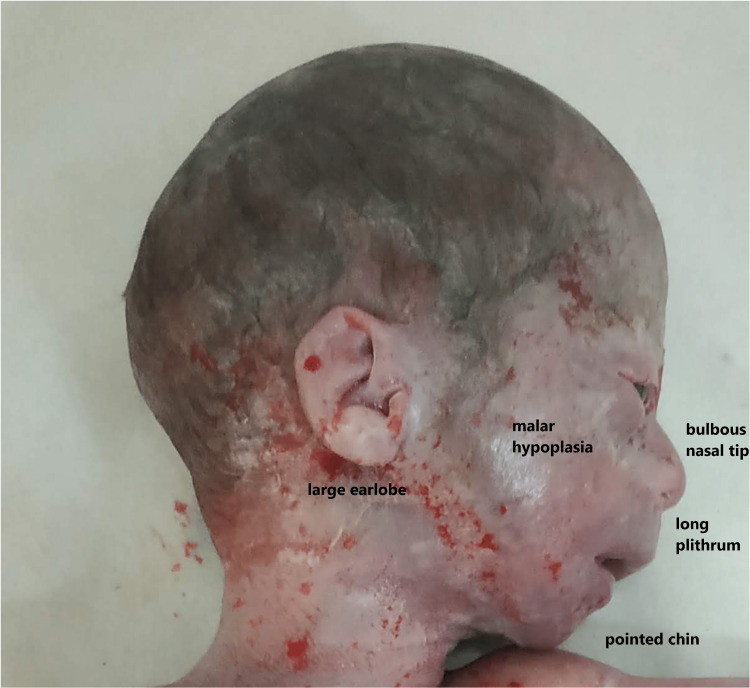
Profile of the affected WBS fetus: facial deformities Personal archive of current case: Nikolaos Tsagkas MD, MSc; Consultant OB/GYN WBS: Williams-Beuren syndrome

In the present case, bulbous nasal tip, long philtrum, malar hypoplasia, pointed chin, and large earlobes were observed in the affected WBS fetus (Figures 1, 2).

Common medical conditions that coexist with WBS include cardiovascular, endocrine, and nervous system abnormalities. Of note, 75% of WBS cases present with clinical cardiac disease, with supravalvular aortic stenosis and pulmonary artery stenosis being the most common findings. Mental retardation, developmental delay, learning disability, and hypotonia are also among the common neurological features of WBS [11]. Furthermore, it has to be mentioned that the differential of hypotonia except for the WBS include T21/DOWN syndrome, T13/PATAU syndrome, Prader-Willi syndrome, Tay-Sachs disease, achondroplasia, and spinal muscular atrophy [16].

In our case, the pregnant woman was routinely offered first-trimester scanning by a fetal medicine specialist as per local practice. At the first trimester scan, the nuchal translucency and the maternal serum screening tests [beta chorionic gonadotropin (bHCG) and pregnancy-associated plasma protein A (PAPPA)] were normal, together with first-trimester anatomical scanning. Fetal crown-rump length (CRL) was showing a small-for-date fetus and, due to the mother being a type 1 diabetic, a 17-week scan was booked to re-evaluate for cardiac anomalies.

During the scan and, specifically when scanning for the anatomy of the digits of the hands, the hand movements seemed to be very reduced for the gestational age. After scanning that lasted more than four hours on two consecutive days, there was a strong, albeit nonspecific, impression of inactivity of the fetal hands and overall hypotonia of the fetus.

Invasive prenatal testing was decided to be performed after counseling, and the repeat scan before termination of the pregnancy at 19 weeks classified the baby at the ninth centile of the growth chart (evidence of intrauterine growth restriction).

As for the genetic procedure, the actual molecular genetic analysis was performed by arrayCGH analysis as mentioned above. In more detail, the microarray used was the “8Χ60Κ G3 ISCA V2” (Agilent Technologies, Santa Clara, CA) and the software Cytogenetics (Agilent Technologies). The specific platform detects imbalances (deletions/duplications) of ~500kbases in the whole genome and imbalances of 50kbases in ~240 genetic regions of known clinical significance, which are related to ~512 genetic conditions according to OMIM. This includes numerical anomalies (trisomies 21, 18, 13), microdeletion syndromes, duplication syndromes, and imbalanced structural chromosomal abnormalities [17].

## Conclusions

Our report introduced a novel scientific question in the field of fetal medicine and prenatal scanning and diagnosis. We described a case of WBS, which, while diagnosed postnatally in a vast majority of cases, was successfully diagnosed in early gestation based on scanning for fetal motility. The question to be addressed in future research is whether fetal inactivity/hypotonia in early gestation is a feature that requires more attention by obstetricians and sonographers (as it can be associated with a spectrum of severe fetal abnormalities) or if the diagnosis of the syndrome in our case was a matter of pure luck?
